# Red ginseng powder fermented with probiotics exerts antidiabetic effects in the streptozotocin-induced mouse diabetes model

**DOI:** 10.1080/13880209.2016.1237978

**Published:** 2016-12-07

**Authors:** Sun-Hee Jang, Jisang Park, Sae-Hae Kim, Kyung-Min Choi, Eun-Sil Ko, Jeong-Dan Cha, Young-Ran Lee, Hyonseok Jang, Yong-Suk Jang

**Affiliations:** a Department of Molecular Biology and the Institute for Molecular Biology and Genetics, Chonbuk National University, Jeonju, Korea;; b Department of Bioactive Material Sciences and Research Center of Bioactive Materials, Chonbuk National University, Jeonju, Korea;; c Department of Efficacy Research, Institute of Jinan Red Ginseng, Jinan, Korea;; d Department of Mathematics, Sogang University, Seoul, Korea;; e Department of Oral Maxillofacial Surgery, Korea University Ansan Hospital, Ansan, Korea

**Keywords:** Diabetes, fermentation, glucose tolerance, insulin

## Abstract

**Context:** Red ginseng (heat-processed *Panax ginseng*) is a well-known alternative medicine with pharmacological antidiabetic activity. It exerts pharmacological effects through the transformation of saponin into metabolites by the intestinal microbiota. Given that intestinal conditions and intestinal microflora vary among individuals, the pharmacological effects of orally administered red ginseng likely may vary among individuals.

**Objective:** To overcome this variation and produce homogeneously effective red ginseng, we evaluated the antidiabetic effects of probiotic-fermented red ginseng in a mouse model.

**Materials and methods:** The antidiabetic efficacy of orally administered probiotic-fermented red ginseng was assessed in ICR mice after induction of diabetes using streptozotocin (170 mg/kg body weight). Samples were given orally for 8 weeks, and indicators involved in diabetic disorders such as body weight change, water intake, blood glucose, glucose tolerance and various biochemical parameters were determined.

**Results:** Oral administration of probiotic-fermented red ginseng significantly decreased the level of blood glucose of about 62.5% in the fasting state and induced a significant increase in glucose tolerance of about 10.2% compared to the control diabetic mice. Additionally, various indicators of diabetes and biochemical data (e.g., blood glycosylated haemoglobin level, serum concentrations of insulin, and α-amylase activity) showed a significant improvement in the diabetic conditions of the mice treated with probiotic-fermented red ginseng in comparison with those of control diabetic mice.

**Discussion and conclusion:** Our results demonstrate the antidiabetic effects of probiotic-fermented red ginseng in the streptozotocin-induced mouse diabetes model and suggest that probiotic-fermented red ginseng may be a uniformly effective red ginseng product.

## Introduction

The incidence of diabetes mellitus, a typical metabolic disease, has been increasing recently. Diabetes is caused by an absolute (type 1 diabetes) or relative (type 2 diabetes) lack of insulin, and the continued hyperglycaemia due to diabetes causes abnormalities in lipid, protein, and sugar metabolism (Kannel & McGee 1979). It also causes various complications, including cardiovascular diseases, decreased renal function, atherosclerosis, blurred vision due to retinal bleeding, foot ulcers and peripheral neuropathies (Amos et al. 1997). Although it is difficult to ‘cure’ diabetes completely, controlling the level of blood glucose is possible. Thus, treating diabetes has focused on preventing diabetic complications and/or suppressing further development of the disease by controlling the level of blood glucose (Amos et al. 1997). Because the ability to fundamentally cure diabetes using insulin or oral hypoglycaemic agents is technically limited and because there are financial burdens as well as the side effects associated with the prolonged intake of medicines, the use of natural products with fewer side effects is strongly recommended. With advances in alternative medicine, there have been many studies on the effects of natural products on antidiabetic activity, and various functional foods for diabetes patients are under development (Oh et al. 2005; Han et al. 2010). In fact, a study conducted in the United States and Australia found that 34.5–48.5% of diabetes patients had used at least one ‘folk’ medicine along with other medications (MacLennan et al. 1996).

Red ginseng, which is acquired by steaming and then drying fresh ginseng *Panax ginseng*, is a functional food known to be effective in various applications. Its ability to improve the post-prandial glucose levels in blood may be especially relevant to diabetes patients (Vuksan et al. 2000, 2008; Sievenpiper et al. 2006). Red ginseng contains various physiological substances, including the ginseng saponins (Sanada et al. 1974; Kimura et al. 1981; Yokozawa et al. 1985; Shin 2010). The saponin components of red ginseng are poorly absorbed into the body directly after administration; they are first converted to metabolites by intestinal microorganisms, such as *Bifidobacterium* spp., *Lactobacillus* spp., and *Saccharomyces* spp., which facilitate the major pharmacological action of red ginseng, although the efficiency of absorption varies depending on body conditions (Trinh et al. 2007; Yang et al. 2007; Liu et al. 2015). Although the metabolic processing of ginseng saponins by microbiota is complicated, and the possibility of further metabolism of fermented red ginseng by intestinal microbiota cannot be excluded, it can be assumed that the biological changes in red ginseng in the anaerobic state are similar to those in the intestines, where intestinal microorganisms use fermentation to transform the components of red ginseng into final metabolites. Furthermore, fermentation of red ginseng may help in producing a uniformly effective product that is readily absorbed in a way that is independent of the individual’s intestinal condition (Kong et al. 2008). Fermentation is one technology capable of improving the physiological activity of natural products. For example, fermentation improves the functionality of green tea (Feng et al. 2002; Kuo et al. 2005; Lee et al. 2012). Additionally, it has been shown that the physiological activities of many natural goods are improved through fermentation (Kusznierewicz et al. 2008; Han et al. 2011). Thus, we prepared a probiotic-fermented red ginseng powder and confirmed its efficacy in diabetes mellitus in the streptozotocin (STZ)-induced diabetes mouse model.

## Materials and methods

### Preparation of probiotic-fermented ginseng powder and experimental materials

A commercial red ginseng extract powder was obtained from Kunbo Inc. (Jinan, Korea). The powder was suspended in water and fermented for 20 days with *Lactobacillus plantarum* (KFCC11611P) at 35–40 °C, then extracts were freeze-dried and administered orally. Qualitative analyses of the ginsenoside composition of the probiotic-fermented red ginseng was performed by high-performance liquid chromatography; changes in the ginsenoside composition before and after fermentation have been described previously (Jang et al. 2016). All chemicals were purchased from Sigma Chemical Co. (St. Louis, MO), unless otherwise specified.

### Experimental animals

Six-week-old male ICR mice were purchased from Orient Bio, Inc. (Sungnam, Korea) and maintained under specific pathogen-free conditions with *ad libitum* access to food and water. Experimental procedures involving laboratory animals were approved by the Institutional Animal Care and Use Committee of the Chonbuk National University (Approval Number: CBU 2015-0004) and followed the guidelines suggested by the committee.

### Diabetes induction

To set up the experimental insulin-dependent diabetes mellitus mouse model, we used the STZ-induced model. Usually, after fasting for 12 h, mice with an average body weight of 30 g were injected intraperitoneally with STZ (170 mg/kg body weight, a dose to induce diabetes in 85% of the treated mice) in 0.1 M citrate buffer (pH 4.0). At 4 days after STZ injection, the mice were fasted for 12 h, and blood was drawn from their tail vein; mice with glucose levels higher than 250 mg/dL were selected as diabetes-induced mice and were randomly assigned to the experimental groups.

### Animal experiments

Experimental animals were divided into five groups with eight mice in each: the untreated group (Normal), the STZ-induced diabetic group (Control), the STZ-induced diabetic group treated with probiotics only (PB), the STZ-induced diabetic group treated with red ginseng (GS), and the STZ-induced diabetic group treated with probiotics-fermented red ginseng (GS + PB). The normal and control groups were given distilled water (DW), whereas the PB group was given 3 × 10^9^ CFU of probiotics per mouse, and the GS and GS + PB groups were given 150 mg/kg body weight of red ginseng and probiotic-fermented red ginseng, respectively. All samples were orally given in 0.4 mL volume once per day for 8 weeks. During the experimental period, body weight and water intake were measured once every 7 and 3 days, respectively.

### Analysis of blood glucose and glucose tolerance levels

Levels of blood glucose were measured once every 2 weeks using a blood glucose strip (Accu-Chek, Roche Diagnostics GmbH, Mannheim, Germany) after a 12 h fasting period. A glucose tolerance test was performed by measuring levels of blood glucose with the blood glucose strip at 15, 30, 45, 60, 90, 120, and 180 min after intraperitoneal administration of glucose solution (2 g/kg body weight) after a 12 h fasting period at the 8th week of the experimental period.

### Analysis of other indicators related to diabetes

To measure the levels of glycated haemoglobin in blood, blood samples were collected at the 8th week of the experiment and injected into a glycated haemoglobin (HbA1c) analytical cartridge (Bio-Rad, Hercules, CA). The level of HbA1c was measured using the Bio-Rad Variant II Turbo system.

Levels of blood insulin were measured using a mouse insulin ELISA kit (ALPCo Diagnostics, Salem, NH) at the 8th week of the experiment with sera prepared from blood drawn from mice fasted for 12 h. The same sera samples were also used in the analysis of α-amylase concentrations using a QuantiChrom α-amylase assay kit (BioAssay Systems, Hayward, CA).

### Biochemical analyses of the sera

To analyze serum components biochemically, samples were collected from the mice in the 8th week of the experiment after a 12-h fasting period. Sample analyses for various indicators, including levels of albumin, total protein, lactate dehydrogenase (LDH), glutamic oxaloacetic transaminase (GOT), glutamic pyruvic transaminase (GPT), creatinine, high-density lipoprotein (HDL), total cholesterol, triglyceride and blood urea nitrogen (BUN), were performed at the Green Cross Reference Lab. (Yongin, Korea).

### Statistical analysis

Statistical analyses were performed using the SPSS software version 16.0 (San Rafael, CA). Results are presented as means ± standard deviations (SDs). To examine the statistical significance of the results, analysis of variance (ANOVA) was performed, and differences were considered statistically significant when *p*-values between groups were <0.05.

## Results

### Oral administration of probiotic-fermented red ginseng is effective in reducing increased water intake in mice with STZ-induced diabetes

**Figure 1.**
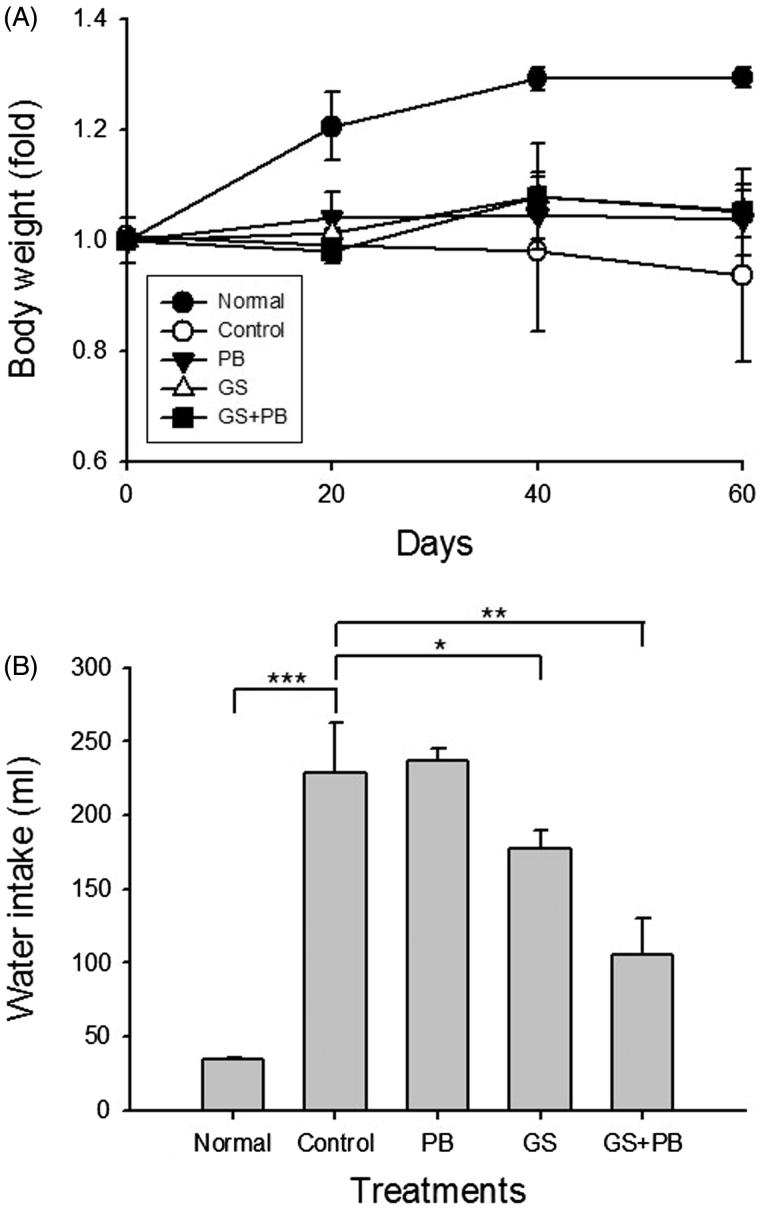
Effects of orally administered probiotic-fermented red ginseng on (A) body weight changes and (B) water intake in STZ-induced diabetic mice. Body weight changes and water intake were measured in normal untreated mice (Normal), STZ-induced diabetic mice (Control), STZ-induced diabetic mice treated with probiotics only (PB), STZ-induced diabetic mice treated with red ginseng (GS), and STZ-induced diabetic group of mice treated with probiotic-fermented red ginseng (GS + PB), as described in the “Materials and methods” section. Data are presented as means ± SD (*n* = 8). *p < 0.05, **p < 0.01 and ***p < 0.001 indicate significant differences.

We observed changes in body weight in the experimental mice three times, once every 20 days after STZ-mediated diabetes induction (Figure 1(A)). Compared with the untreated group (Normal) of mice, which showed continued increases in body weight, the mice treated with probiotic alone (PB) had unchanged and consistent body weights. In contrast, the body weights of the mice treated with probiotic-fermented red ginseng (GS + PB) decreased slightly at the first measurement and quickly recovered to levels comparable to those of the PB group. However, the STZ-induced diabetes group (Control) showed decreased body weights that were about one-third of those of the normal group on day 60 after diabetes induction.

We next measured the amount of water intake, because increased water intake is a physiological characteristic of diabetes. As expected in a typical diabetic state, in the 8th week of the experiment, the amount of water consumed by a mouse in 5 days was increased significantly (*p* < 0.001), by ∼9-fold compared with that of normal untreated mice, in the control diabetic group. The amount of water intake in the GS group of mice was significantly lower (*p* < 0.05) than that in the control diabetic group. More importantly, the amount of water intake in the GS + PB group was significantly lower to a large degree (*p* < 0.01) than in the GS group (Figure 1(B)).

### Oral administration of probiotic-fermented red ginseng is effective in reducing increased levels of blood glucose and enhancing low glucose tolerance in the diabetic group of mice

**Figure 2.**
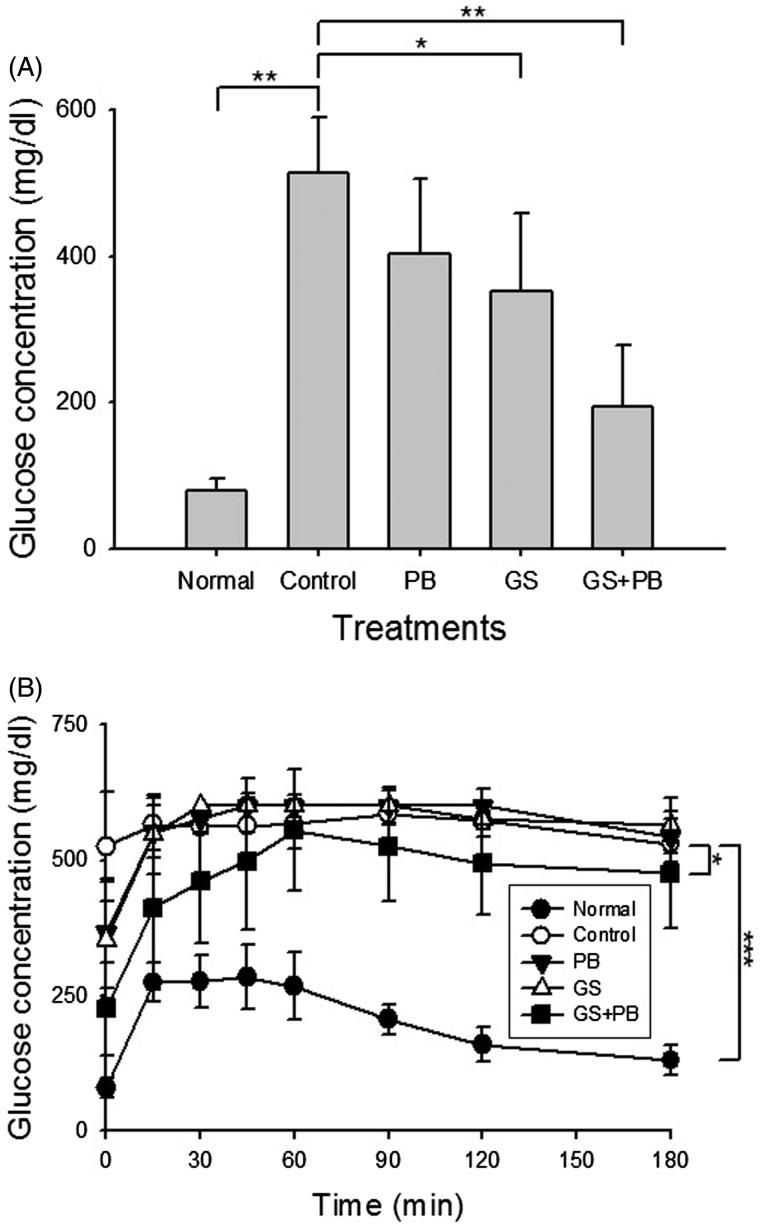
Effects of orally administered probiotic-fermented red ginseng on levels of blood glucose (A) at the 8th week of diabetes induction and (B) after intraperitoneal glucose injection at the 8th week of diabetes induction in the STZ-induced diabetic mice. Blood glucose levels were measured in normal untreated mice (Normal), STZ-induced diabetic mice (Control), STZ-induced diabetic mice treated with probiotics only (PB), STZ-induced diabetic mice treated with red ginseng (GS), and STZ-induced diabetic mice treated with probiotic-fermented red ginseng (GS + PB). Data are presented as means ± SD (*n* = 8). *p < 0.05, **p < 0.01, and ***p < 0.001 indicate significant differences.

Blood glucose level is the most important indicator of diabetic status. To assess the effects of oral administration of GS + PB on diabetes, the level of blood glucose was determined (Figure 2(A)). The blood glucose level in the GS + PB group of mice was significantly lower (198.0 ± 85.6 mg/dL; *p* < 0.01) compared with the increased blood glucose level (515.0 ± 74.1 mg/dL) observed in the control diabetic group of mice. In contrast, although it was relatively lower than that in the control diabetic group of mice, the blood glucose level of the PB group of mice was not reduced significantly.

We next measured how efficiently the high concentration of intraperitoneally introduced glucose was cleared from the blood (Figure 2(B)). Although blood glucose increased rapidly at 30 min after glucose injection in the normal group of mice, the level was significantly lower than that in control diabetic mice, and the level decreased rapidly to background levels. All mice, in which diabetes was induced, showed inefficient blood glucose clearance compared with the normal group. Importantly, blood glucose levels measured at 3 h after glucose injection were significantly lower (*p* < 0.05) in the GS + PB group of mice versus in the control diabetic mice. However, blood glucose levels in the PB group of mice was similar to that in the control diabetic group during the test period.

### Oral administration of probiotic-fermented red ginseng is effective in reducing increased levels of HbA1c

**Figure 3.**
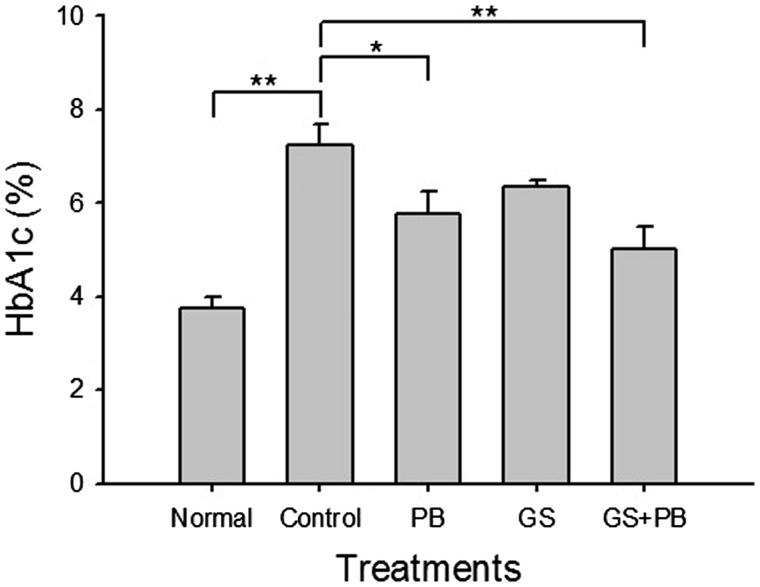
Effects of orally administered probiotic-fermented red ginseng on levels of blood HbA1c at the 8th week after diabetes induction. The level of HbA1c was measured in normal untreated mice (Normal), STZ-induced diabetic mice (Control), STZ-induced diabetic mice treated with probiotics only (PB), STZ-induced diabetic mice treated with red ginseng (GS), and STZ-induced diabetic mice treated with probiotic-fermented red ginseng (GS + PB). Data are presented as means ± SD (*n* = 4). *p < 0.05 and **p < 0.01 indicate significant differences.

HbA1c represents glycated haemoglobin and the level of HbA1c increases when blood glucose level increases. We measured levels of HbA1c in the blood to assess the effects of GS + PB administration (Figure 3). The HbA1c blood level was increased significantly (*p* < 0.01) in the control diabetic mice; in comparison, this level was significantly reduced (*p* < 0.01) in the GS + PB-treated group. However, the PB-only-treated group of mice also showed significantly (*p* < 0.05) reduced level of HbA1c compared with the control diabetic group of mice.

### Oral administration of probiotic-fermented red ginseng is effective in restoring blood insulin levels and α-amylase activity

**Figure 4.**
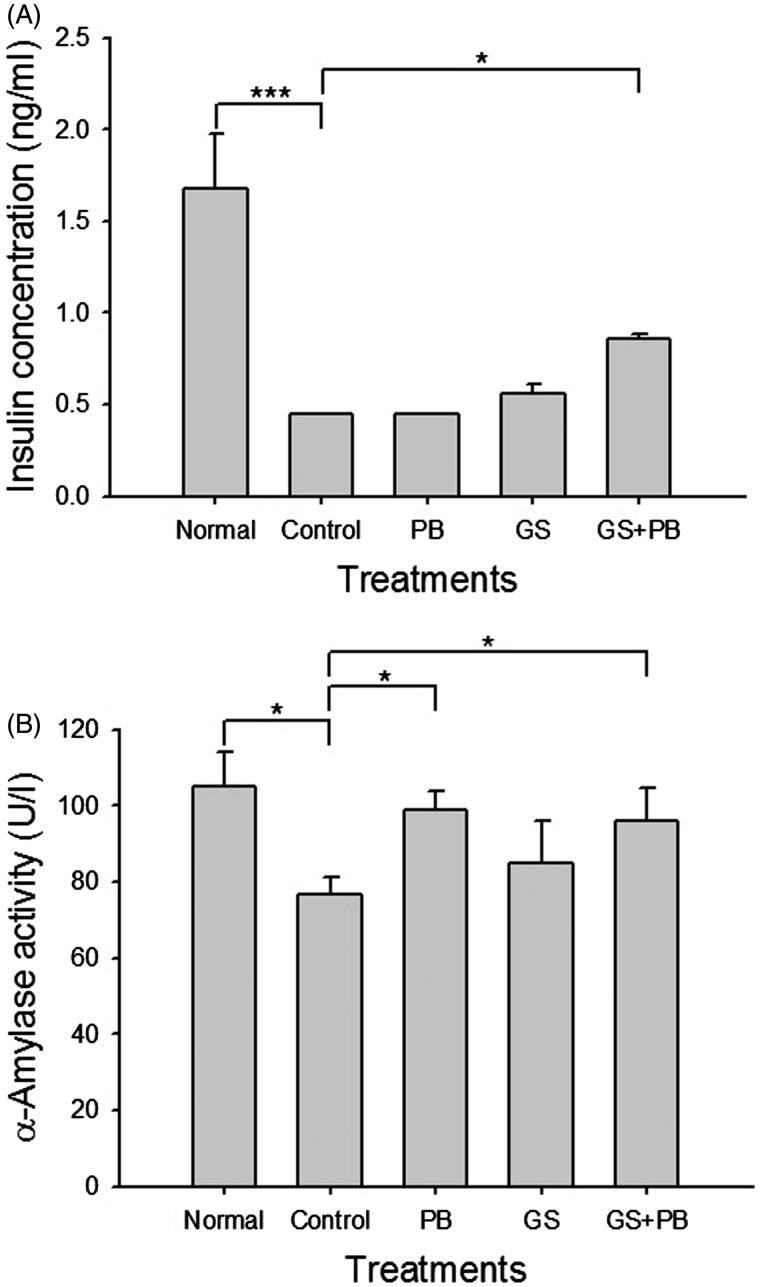
Effects of orally administered probiotic-fermented red ginseng on levels of (A) blood insulin and (B) blood α-amylase activity at the 8th week after diabetes induction. Blood was drawn from the normal untreated mice (Normal), STZ-induced diabetic mice (Control), STZ-induced diabetic mice treated with probiotics only (PB), STZ-induced diabetic mice treated with red ginseng (GS), and STZ-induced diabetic mice treated with probiotic-fermented red ginseng (GS + PB); levels of insulin and α-amylase activity were measured as described in the “Materials and methods” section. Data are presented as means ± SD (*n* = 4). *p < 0.01 and ***p < 0.001 indicate significant differences.

We next assessed the effects of the oral administration of GS + PB on the levels of blood insulin (Figure 4(A)). As shown in the figure, the level of blood insulin in the control diabetic group was significantly lower (*p* < 0.001) than it was in the normal untreated mice; this result was expected, because STZ treatment to induce diabetes is known to exert toxic effects on insulin-producing β-cells. Importantly, although oral administration of GS + PB significantly increased the levels of blood insulin (*p* < 0.05), its ability to completely restore such levels seemed somewhat limited, which might have been caused by STZ-mediated permanent damage to β-cells.

It is known that a low serum α-amylase level is associated with diabetes and that insulin restores the level of α-amylase (Kim et al. 1990; Lee et al. 2011). The level of blood α-amylase in control diabetic mice was significantly (*p* < 0.05) lower than that in the normal untreated mice (Figure 4(B)). Additionally, oral administration of GS + PB restored the level of α-amylase to a significant extent (*p* < 0.05).

### Oral administration of probiotic-fermented red ginseng is effective in restoring various biochemical indicators associated with diabetes

**Figure 5.**
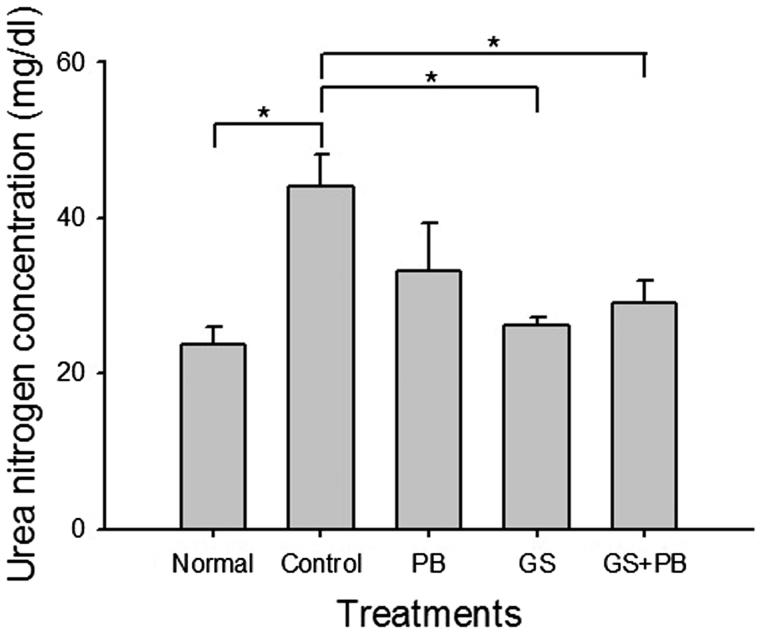
Effects of orally administered probiotic-fermented red ginseng on levels of blood urea nitrogen (BUN) at the 8th week after diabetes induction. Blood was drawn from normal untreated mice (Normal), STZ-induced diabetic mice (Control), STZ-induced diabetic mice treated with probiotics only (PB), STZ-induced diabetic mice treated with red ginseng (GS), and STZ-induced diabetic group of mice treated with probiotic-fermented red ginseng (GS + PB); levels of BUN were determined as described in the “Materials and methods” section. Data are presented as means ± SD (*n* = 4). *p < 0.05 indicates significant differences.

We next performed biochemical analysis on various parameters, including albumin, total protein, LDH, GOT, GPT, creatinine, HDL, total cholesterol, and triglycerides. The level of GOT in the control diabetic mice (874 ± 128 U/L) was significantly higher (*p* < 0.05) than in the normal untreated group of mice (606 ± 120 U/L); the latter level was significantly higher (*p* < 0.01) than it was in the GS + PB-treated group of mice (371 ± 143 U/L). Similarly, the GPT level in the control diabetic group of mice (725 ± 395 U/L) was significantly higher (*p* < 0.05) than that in the normal untreated mice (81 ± 6 U/L); the latter was significantly higher (*p* < 0.05) than that in the GS + PB-treated mice (170 ± 128 U/L). Most other indicators that were changed in the control diabetic mice in comparison with the normal untreated mice were restored to levels similar to those in the normal mice after oral administration of GS + PB (data not shown). Finally, we performed a BUN accumulation experiment, as BUN is an indicator of renal function (Figure 5). The BUN blood levels in control diabetic mice were significantly higher (*p* < 0.05) than those in the normal untreated mice; the BUN level was significantly (*p* < 0.05) decreased in the groups that received oral GS and GS + PB, whereas there was no significant difference in the PB-only-treated mice.

## Discussion

STZ, a nitrosourea alkylating agent that has been used as a diabetes inducer (Evans et al. 1965; Junod et al. 1967), selectively destroys β-cells, which, in turn, decreases the level of insulin in the body and inhibits normal sugar metabolism, resulting in the characteristic symptoms of hyperglycaemia. We used STZ to induce type I diabetes in mice and assessed the functioning of β-cells in STZ-mediated diabetes-induced mice by measuring body weight, water intake, blood glucose, glucose tolerance, blood serum insulin, α-amylase and various biochemical indicators. Additionally, the influence of oral administration of probiotic-fermented red ginseng (GS + PB) on these indicators was assessed to confirm the antidiabetic effect of this substance, including its ability to produce a uniformly effective red ginseng product with a homogenous efficacy that is not influenced by each individual’s intestinal microbiota.

Previous studies on the suppressive function of ginseng saponin on hyperglycaemia suggested that ginseng saponin worked on glycogen oxidation in the liver and muscles and that partly refined saponin was involved in various metabolic processes related to fat and sugar metabolism in normal mice. These studies also reported that a diet with cultured ginseng powder lowers blood glucose levels in STZ-induced diabetic mice (Ng & Yeung 1985; Lee et al. 1997, 2003). Our current study confirmed that GS + PB effectively lowered blood glucose levels in the STZ-induced diabetes animal model, suggesting that, similar to red ginseng, this substance may be useful as an anti-hyperglycaemic product (Figure 2(A)). Glucose tolerance tests also confirmed the efficacy of GS + PB as an anti-hyperglycaemic product (Figure 2(B)). Moreover, data regarding the suppression of HbA1c blood levels indicate the long-term effectiveness of this treatment of hyperglycaemia given that such levels reflect the progression of diabetes over a 1–3-month period (Figure 3). Additionally, it has been reported that a 1% reduction in HbA1c decreases the occurrence of diabetic complications, such as myocardial infarction, by 14% and that of microvessel disease, such as retinopathy, by 37% (Stratton et al. 2000). Thus, it can be concluded that long-term blood glucose control is possible through taking GS + PB to reduce the dangers of diabetic complications.

The most important hormone in controlling sugar metabolism is insulin, which is produced by β-cells. Additionally, in diabetes, the mRNA available for α-amylase production in the exocrine acini of the colon decreases, thereby decreasing the synthesis of α-amylase; it has been reported that injection of insulin into diabetic mice increases the colonic excretion of α-amylase (Williams & Goldfine 1985). We observed that the level of insulin, which controls blood sugar levels, was increased significantly in the group receiving oral administration of GS + PB in comparison with in the control diabetic group (Figure 4(A)). Moreover, α-amylase secretion was increased significantly in the group receiving the oral administration of GS + PB compared with in the control diabetic group (Figure 4(B)). These results suggest that GS + PB positively affects the production of insulin by stimulating β-cells in the colon and improves sugar metabolism in peripheral tissues, thereby causing a sharp decline in blood glucose levels.

The main pharmaceutical component of ginseng is saponin, which is also known as ginsenoside. However, ginseng also has various non-saponin pharmacological components including acidic polysaccharides, polyacetylenes such as panaxydol, and organic compounds such as maltol. Parts of these components undergo chemical changes during the manufacturing process, generating saponin and other bioactive components (Christensen 2009). The saponin in ginseng is mostly malonyl-ginsenoside, and malonic acid is released from malonyl-ginsenoside, leaving trace saponins including Rg3, Rg2, Rh2, Rs1, Rs2 and Rh4. Although red ginseng goes through the same pharmacological changes and is not entirely different from ginseng, some reports have shown that the efficacy of red ginseng for enhancing blood circulation, cancer suppression, and defence against various infections is superior to that of ginseng (Sung et al. 2009; Nam 2005). Interestingly, fermentation of red ginseng with probiotics reduces the total concentration of ginsenosides about 30%, suggesting that the fermentation process may aid the absorption of red ginseng by the intestine (Jang et al. 2016). Notably, the concentration of Rg3, which is a crucial component in the antidiabetic activity of red ginseng, increased about 3-fold after fermentation (Kang et al. 2013; Kim et al. 2015).

## Conclusion

This study confirmed that GS + PB has an antidiabetic effect, as the results of our experiments confirmed improvements in the typical symptoms of diabetes, including increases in blood glucose levels, body weight loss, increased HbA1c, and decreased insulin density and α-amylase activity, in STZ-induced diabetic mice. Additionally, the experiments showed that oral administration of GS + PB improved serum lipid levels and decreased levels of BUN in the blood. GS + PB may be particularly effective in decelerating the progression toward heart failure caused by diabetes by preventing the accumulation of BUN. Taken together, our results suggest that GS + PB may be useful for alleviating diabetic symptoms and can be used as a uniformly effective red ginseng product.

## References

[CIT0001] Amos AF, McCarty DJ, Zimmet P. 1997 The rising global burden of diabetes and its complications: estimates and projections to the year 2010. Diabet Med. 14:S1–S85. 9450510

[CIT0002] Christensen LP. 2009 Ginsenosides chemistry, biosynthesis, analysis, and potential health effects. Adv Food Nutr Res. 55:1–99. 18772102 10.1016/S1043-4526(08)00401-4

[CIT0003] Evans JS, Gerritsen GC, Mann KM, Owen SP. 1965 Antitumor and hyperglycemic activity of streptozotocin (NSC-37917) and its cofactor, U-15,774. Cancer Chemother Rep. 48:1–6. 4953644

[CIT0004] Feng Q, Torii Y, Uchida K, Nakamura Y, Hara Y, Osawa T. 2002 Black tea polyphenols, theaflavins, prevent cellular DNA damage by inhibiting oxidative stress and suppressing cytochrome P450 1A1 in cell cultures. J Agr Food Chem. 50:213–220. 11754570 10.1021/jf010875c

[CIT0005] Han HK, Je HS, Kim GH. 2010 Effect of *Cirsium japonicum* powder on plasma glucose and lipid level in streptozotocin induced diabetic rats. Korean J Food Sci Technol. 42:343–349.

[CIT0006] Han CC, Wei H, Guo J. 2011 Anti-inflammatory effects of fermented and non-fermented *Sophora flavescens*: a comparative study. BMC Complement Altern Med. 11:100–106. 22026927 10.1186/1472-6882-11-100 PMC3215180

[CIT0007] Jang SH, Park J, Kim SH, Choi KM, Ko ES, Cha JD, Lee YR, Jang H, Jang YS. 2016 Oral administration of red ginseng powder fermented with probiotic alleviates the severity of dextran-sulfate sodium-induced colitis in a mouse model. Chin J Nat Med. In press. 10.1016/S1875-5364(17)30035-3 28411687

[CIT0008] Junod A, Lambert AE, Orci L, Pictet R, Gonet AE, Renold AE. 1967 Studies of the diabetogenic action of streptozotocin. Proc Soc Exp Biol Med. 126:201–205. 4864021 10.3181/00379727-126-32401

[CIT0009] Kang KS, Ham J, Kim YJ, Park JH, Cho EJ, Yamabe N. 2013 Heat-processed *Panax ginseng* and diabetic renal damage: active components and action mechanism. J Ginseng Res. 37:379–388. 24233065 10.5142/jgr.2013.37.379 PMC3825853

[CIT0010] Kannel WB, McGee DL. 1979 Diabetes and cardiovascular disease. The Framingham study. JAMA. 241:2035–2038. 430798 10.1001/jama.241.19.2035

[CIT0011] Kim SK, Cuzzort LM, McKean RK, Allen ED. 1990 Effects of diabetes and insulin on alpha-amylase messenger RNA levels in rat parotid glands. J Dent Res. 69:1500–1504. 2143513 10.1177/00220345900690081001

[CIT0012] Kim KS, Yang HJ, Lee IS, Kim KH, Park J, Jeong HS, Kim Y, Ahn KS, Na YC, Jang HJ. 2015 The aglycone of ginsenoside Rg3 enables glucagon-like peptide-1 secretion in enteroendocrine cells and alleviates hyperglycemia in type 2 diabetic mice. Sci Rep. 5:18325. 26675132 10.1038/srep18325 PMC4682129

[CIT0013] Kimura M, Waki I, Chujo T, Kikuchi T, Hiyama C, Yamazaki K, Tanaka O. 1981 Effects of hypoglycemic components in ginseng radix on blood insulin level in alloxan diabetic mice and on insulin release from perfused rat pancreas. J Pharmacobiodyn. 4:410–417. 7026762 10.1248/bpb1978.4.410

[CIT0014] Kong BM, Park MJ, Min JW, Kim HB, Kim SH, Kim SY, Yang DC. 2008 Physicochemical characteristics of white, fermented and red ginseng extracts. J Ginseng Res. 32:238–243.

[CIT0015] Kuo KL, Weng MS, Chiang CT, Tsai YJ, Lin-Shiau SY, Lin JK. 2005 Comparative studies on the hypolipidemic and growth suppressive effects of oolong, black, pu-erh, and green tea leaves in rats. J Agric Food Chem. 53:480–489. 15656692 10.1021/jf049375k

[CIT0016] Kusznierewicz B, Śmiechowska A, Bartoszek A, Namieśnik J. 2008 The effect of heating and fermenting on antioxidant properties of white cabbage. Food Chem. 108:853–861. 26065745 10.1016/j.foodchem.2007.11.049

[CIT0017] Lee HA, Kwon SO, Lee HB. 1997 Hypoglycemic action of components from red ginseng: (I) Investigation of the effect of ginsenosides from red ginseng on enzymes related to glucose metabolism in cultured rat hepatocytes. Korean J Ginseng Sci. 21:174–186.

[CIT0018] Lee IS, Lee SO, Lee IZ. 2003 Effect of tissue cultured ginseng on blood glucose and lipids in streptozotocin-induced diabetic rats. Korean J Food Sci Technol. 35:280–285.

[CIT0019] Lee JG, Park SW, Cho BM, Lee S, Kim YJ, Jeong DW, Yi YH, Cho YH. 2011 Serum amylase and risk of the metabolic syndrome in Korean adults. Clin Chim Acta. 412:1848–1853. 21726545 10.1016/j.cca.2011.06.023

[CIT0020] Lee SI, Lee YK, Kim SD, Yang SH, Suh JW. 2012 Dietary effects of post-fermented green tea by *Monascus pilosus* on the body weight, serum lipid profiles and the activities of hepatic antioxidative enzymes in mouse fed a high fat diet. J Appl Biol Chem. 55:85–94.

[CIT0021] Liu C, Hu M, Guo H, Zhang M, Zhang J, Li F, Zhong Z, Chen Y, Li Y, Xu P, et al 2015 Combined contribution of increased intestinal permeability and inhibited deglycosylation of ginsenoside Rb1 in the intestinal tract to the enhancement of ginsenoside Rb1 exposure in diabetic rats after oral administration. Drug Metab Dispos. 43:1702–1710. 26265741 10.1124/dmd.115.064881

[CIT0022] MacLennan AH, Wilson DH, Taylor AW. 1996 Prevalence and cost of alternative medicine in Australia. Lancet. 347:569–573. 8596318 10.1016/s0140-6736(96)91271-4

[CIT0023] Nam KY. 2005 The comparative understanding between red ginseng and white ginsengs, processed ginsengs (*Panax ginseng* C. A. Meyer). J Ginseng Res. 29:1–18.

[CIT0024] Ng TB, Yeung HW. 1985 Hypoglycemic constituents of *Panax ginseng*. Gen Pharmacol. 16:549–552. 3910515 10.1016/0306-3623(85)90140-5

[CIT0025] Oh WK, Lee CH, Lee MS, Bae EY, Sohn CB, Oh H, Kim BY, Ahn JS. 2005 Antidiabetic effects of extracts from *Psidium guajava*. J Ethnopharmacol. 96:411–415. 15619559 10.1016/j.jep.2004.09.041

[CIT0026] Sanada S, Kondo N, Shoji J, Tanaka O, Shibata S. 1974 Studies on the saponins of ginseng. I. Structures of ginsenoside-R_0_, -Rb_1_, -Rb_2_, -Rc and -Rd. Chem Pharm Bull (Tokyo). 22:421–428.

[CIT0027] Shin YS. 2010 Comparisons of ginsenosides and anti-inflammatory effects of white ginseng and puffed red ginseng. Korean J Food Cook Sci. 26:475–480.

[CIT0028] Sievenpiper JL, Sung MK, Di Buono M, Lee KS, Nam KY, Arnason JT, Leiter LA, Vuksan V. 2006 Korean red ginseng rootlets decrease acute postprandial glycemia: results from sequential preparation- and dose-finding studies. J Am Coll Nutr. 25:100–107. 16582025 10.1080/07315724.2006.10719519

[CIT0029] Stratton IM, Adler AI, Neil HAW, Matthews DR, Manley SE, Cull CA, Hadden D, Turner RC, Holman RR., 2000 Association of glycemia with macrovascular and microvascular complications of type 2 diabetes (UKPDS 35): prospective observational study. BMJ. 321:405–412. 10938048 10.1136/bmj.321.7258.405 PMC27454

[CIT0030] Sung H, Jung YS, Cho YK. 2009 Beneficial effects of a combination of Korean red ginseng and highly active antiretroviral therapy in human immunodeficiency virus type 1-infected patients. Clin Vaccine Immunol. 16:1127–1131. 19535541 10.1128/CVI.00013-09 PMC2725544

[CIT0031] Trinh HT, Han SJ, Kim SW, Lee YC, Kim DH. 2007 *Bifidus* fermentation increases hypolipidemic and hypoglycemic effects of red ginseng. J Microbiol Biotechnol. 17:1127–1133. 18051323

[CIT0032] Vuksan V, Stavro MP, Sievenpiper JL, Koo VY, Wong E, Beljan-Zdravkovic U, Francis T, Jenkins AL, Leiter LA, Josse RG, et al 2000 American ginseng improves glycemia in individuals with normal glucose tolerance: effect of dose and time escalation. J Am Coll Nutr. 19:738–744. 11194526 10.1080/07315724.2000.10718073

[CIT0033] Vuksan V, Sung MK, Sievenpiper JL, Stavro PM, Jenkins AL, Di Buono M, Lee KS, Leiter LA, Nam KY, Arnason JT, et al 2008 Korean red ginseng (*Panax ginseng*) improves glucose and insulin regulation in well-controlled, type 2 diabetes: Results of a randomized, double-blind, placebo-controlled study of efficacy and safety. Nutr Metab Cardiovasc Dis. 18:46–56. 16860976 10.1016/j.numecd.2006.04.003

[CIT0034] Williams JA, Goldfine ID. 1985 The insulin-pancreatic acinar axis. Diabetes. 34:980–986. 2412919 10.2337/diab.34.10.980

[CIT0035] Yang CS, Ko SR, Cho BG, Lee JY, Kim KH, Shin DM, Yuk JM, Sohn HJ, Kim YS, Wee JJ, et al 2007 Compound K (CK) rich fractions from Korean red ginseng inhibit Toll-like receptor (TLR) 4- or TLR9-mediated mitogen-activated protein kinases activation and pro-inflammatory responses in murine macrophages. J Ginseng Res. 31:181–190.

[CIT0036] Yokozawa T, Kobayashi T, Oura H, Kawashima Y. 1985 Studies on the mechanism of the hypoglycemic activity of ginsenoside-Rb2 in streptozotocin-diabetic rats. Chem Pharm Bull (Tokyo). 33:869–872. 4017130 10.1248/cpb.33.869

